# State of the Art in Parent-Delivered Pain-Relieving Interventions in Neonatal Care: A Scoping Review

**DOI:** 10.3389/fped.2021.651846

**Published:** 2021-04-27

**Authors:** Alexandra Ullsten, Matilda Andreasson, Mats Eriksson

**Affiliations:** ^1^Center for Clinical Research, Region Värmland, Karlstad, Sweden; ^2^Faculty of Medicine and Health, School of Health Sciences, Örebro University, Örebro, Sweden; ^3^Faculty of Medicine and Health, School of Medical Sciences, Örebro University, Örebro, Sweden

**Keywords:** newborn infant, pain, pain management, parent, parent-delivered interventions, scoping review

## Abstract

**Introduction:** Parents' active involvement during painful procedures is considered a critical first step in improving neonatal pain practices. Of the non-pharmacological approaches in use, the biopsychosocial perspective supports parent-delivered interventions, in which parents themselves mediate pain relief, consistent with modern family-integrated care. This scoping review synthesizes the available research to provide an overview of the state of the art in parent-delivered pain-relieving interventions.

**Methods:** A scoping review was performed to achieve a broad understanding of the current level of evidence and uptake of parent-driven pain- and stress-relieving interventions in neonatal care.

**Results:** There is a strong evidence for the efficacy of skin-to-skin contact and breastfeeding, preferably in combination. These parent-delivered interventions are safe, valid, and ready for prompt introduction in infants' pain care globally. Research into parents' motivations for, and experiences of, alleviating infant pain is scarce. More research on combined parent-delivered pain alleviation, including relationship-based interventions such as the parent's musical presence, is needed to advance infant pain care. Guidelines need to be updated to include infant pain management, parent-delivered interventions, and the synergistic effects of combining these interventions and to address parent involvement in low-income and low-tech settings.

**Conclusions:** A knowledge-to-practice gap currently remains in parent-delivered pain management for infants' procedure-related pain. This scoping review highlights the many advantages of involving parents in pain management for the benefit not only of the infant and parent but also of health care.

## Introduction

“It's time to put children at the heart of our vision for a sustainable humanity,” the Lancet proclaimed in the beginning of 2020, when they introduced a special science-based campaign across their journals focusing on child and adolescent health and well-being (1). In October, 2020, the Lancet Child & Adolescent Health Commission published their report stating, “It is time for change.” The commission presented four transformative goals for research and clinical practice to advance the field of pediatric pain over the next 10 years; make pain matter, make pain understood, make pain visible, and make pain better (2). At the heart of this scoping review, we put the critically ill and vulnerable hospitalized infants who suffer the most from repeated, cumulative, and inadequately treated procedural pain in addition to separation from their parents. In line with the Lancet's goals and visions, this scoping review acknowledges the important international appeal to make infants' needs and parents' views visible in order to make procedural pain better. While focusing on the parent as a compassionate and well-informed deliverer of pain relief, this review aims to advance current research on parent-delivered pain-relieving interventions in neonatal care.

Undertreated, unrecognized, or poorly managed pain in infancy puts an individual at risk of severe short-term (3) and long-lasting (4) negative consequences such as chronic pain that continue into adulthood (2). Sufficient pain prevention and treatment are cornerstones of family-centered neonatal care, and parents are essential for improving the treatment of neonatal pain. Parents cannot only provide valuable information about their infant's pain experience but also protect their infants during painful procedures by blunting their painful effects (5). The goal of the caregiving system is to increase parent–infant proximity to protect the infant. Positive parent–infant interactions have been demonstrated to buffer the connections between early neonatal pain in preterm infants and their subsequent cognitive functioning and mental health outcomes (6). However, parents of infants admitted to neonatal care experience a substantial amount of stress (7). One of the most stressful experiences for parents in the neonatal unit, along with the loss of their parental role, is the worry that their infant will suffer pain (8–10). These stressors are associated with higher stress levels in parents, which in turn may impact upon healthy attachment and bonding, aspects that are vital for the long-term development of the infant. Parents' active involvement during painful procedures is considered a critical first step in improving neonatal pain practices (10). In addition to better outcomes for their infants, parents who support their infants during medical procedures can also benefit themselves. Feeling they are helping and protecting their infant can contribute to parents' sense of control in a challenging situation (11) and affirm their parental role (12, 13). Parents who are present during painful procedures report lower distress and more satisfaction with care (9) and feel empowered in their caregiving role (14). Parents need and want to participate actively in their infant's pain management, and they should be educated and guided through various means, not just verbal information, to mitigate their infant's pain (15–22). Coaching parents to better meet their infant's attachment needs during times of pain may lead to more efficacious interventions (23).

This scoping review synthesizes the available research evidence to provide an overview of the state of the art in parent-delivered pain-relieving interventions. Pain research needs to include the whole biopsychosocial model advancing the knowledge of multiple treatment options in all areas of psychological, pharmacological, and physical interventions (2, 24). The biopsychosocial perspective strongly supports parent-delivered interventions (17, 25). In parent-delivered psychophysical interventions, the parents themselves mediate their infant's pain relief (25). Parent-delivered pain alleviation is consistent with modern family-centered care, in which the best interests of the infant and family are put ahead of the staff's convenience (25). Examples of such biopsychosocial interventions are skin-to-skin contact (SSC) (26), breastfeeding (27), live parental infant-directed singing (17), facilitated tucking (28), and holding (29). Few studies have been published on the efficacy of combined multisensorial parent-delivered interventions. So far, research shows that combined parent-delivered pain management such as SSC along with breastfeeding is more effective in reducing infants' responses to pain than either intervention alone (30). Growing evidence supports the impact of parents' active involvement in pain alleviation (31). However, there currently remains a knowledge-to-practice gap in parent-delivered management of infants' procedure-related pain. Little is known of the extent to which parent-delivered pain management is recommended and used in clinical guidelines or how parents experience being the deliverers of pain relief.

## Objectives

The overall purpose of this scoping review is to identify, characterize, and summarize research evidence on parent-delivered pain-relieving interventions in neonatal care where the parents themselves deliver the pain management, as well as highlight current knowledge gaps and research priorities [cf. Peters et al. (32)]. This scoping review may provide the basis for informing current and future policy and practice as well as research in parent-delivered neonatal pain management.

Specific objectives are as follows:

1. Explore the breadth and extent of the literature, identify the types of available evidence, map and summarize the evidence, and inform future research on parent-delivered pain- and stress-relieving interventions in neonatal care.

2. Describe parents' experiences of delivering pain and pain-related stress relief to their newborn infant.

3. Map and summarize recommendations as well as define knowledge gaps in national and international guidelines and in professional organizations or networks.

## Methods

A scoping review was concluded to be the most appropriate to provide a broad overview of the evidence on parent-delivered pain- and stress-relieving interventions in neonatal care. A scoping review would also map the extent and diversity including knowledge gaps of the evidence and knowledge available from research papers and policy documents that guide practice in the field, as well as highlight where more research is warranted (32).

An *a priori* review protocol was published predefining the objectives, methods, inclusion and exclusion criteria, data extraction procedure, and data analysis allowing for transparency of the scoping review process (https://zenodo.org/record/3787492#.YBaaUS2HK-s). No major deviations of the scoping review from the protocol occurred. The scoping review process followed the nine-stage scoping review framework outlined by Peters et al. (32).

### Search Strategy and Databases

Balancing feasibility with breadth and comprehensiveness, searches were performed in CINAHL, Embase, Joanna Briggs Institute EBP Database, Medline, and PsycInfo. The search strategy sought to identify both quantitative and qualitative studies including published conference abstracts, guidelines, and policy documents. The reference list of all identified reports, articles, and systematic reviews was manually searched for additional studies. The authors' expertise in the research area was also used in the manual search. Search terms and the full search syntax can be found in the published *a priori* review protocol (https://zenodo.org/record/3787492#.YBaaUS2HK-s).

### Search Terms

Search terms were text words and MeSH terms, depending on the databases. The following terms were used, combined with AND or OR and in full or truncated versions: Family; Family-cent(e)red; Family nursing; Father(s); Infant, Newborn; Intensive Care; Involvement; Breastfeeding; Kangaroo-mother care method; Maternal; Maternal behavior; Mother(s); Music; Music therapy; Neonatal nursing; Pain; Pain management; Parent-child relations; Parenting; Parents; Paternal; Paternal behavior; Physiologic; Physical reaction; Response; Singing; Single parent; Skin to skin; Stress; Song; Tactual perception; Touch; Vocal; and Voice.

### Identify Relevant Studies

Studies were included based on the following criteria:

- Human studies in English or Nordic languages on infants aged ≤ 1 month.
- Primary research with quantitative and/or qualitative designs including published conference abstracts, guidelines, standards, and policy documents.
- Studies with descriptions of parent-delivered pain- and stress-relieving intervention in newborn care irrespective of medical setting and descriptions of parents' experiences of delivering pain and stress relief to their newborn infant in newborn care irrespective of medical setting.
- Guidelines and recommendations based on consensus group methods or equivalent, for parent-delivered interventions in newborn care issued by a national or regional health authority or a professional healthcare organization or network,
- Secondary research such as systematic reviews was only retrieved for the manual searches of literature and studies and to inform the introduction and discussion part of the scoping review.

Studies were excluded based on the following criteria:

- Studies unavailable in English or Nordic languages.
- Studies on pain in mothers (e.g., during labor or postop), with older children (over 1 month), and animal studies.
- Since the use of statistical meta-analysis or meta-synthesis is typically not conducted in a scoping review, peer-reviewed literature was determined as the basic criteria for the included evidence in this review. Evidence types including unpublished and ongoing trials, dissertations, and conference proceedings were consequently excluded.
- Secondary research such as systematic and other sorts of reviews were not included in the results. However, individual studies from identified reviews were included if relevant.
- Conference abstracts were excluded if they did not present unambiguous methods and results. Guidelines were excluded if they were not issued by a national or regional health authority or a professional healthcare organization or network.
- The database searches were limited to papers published in the years 2010–2020. However, manual searches in systematic reviews also identified relevant primary research between 2000 and 2010 (e.g., parent-delivered interventions with SSC). These were added manually.

### Selecting the Evidence

**Figure 1:**
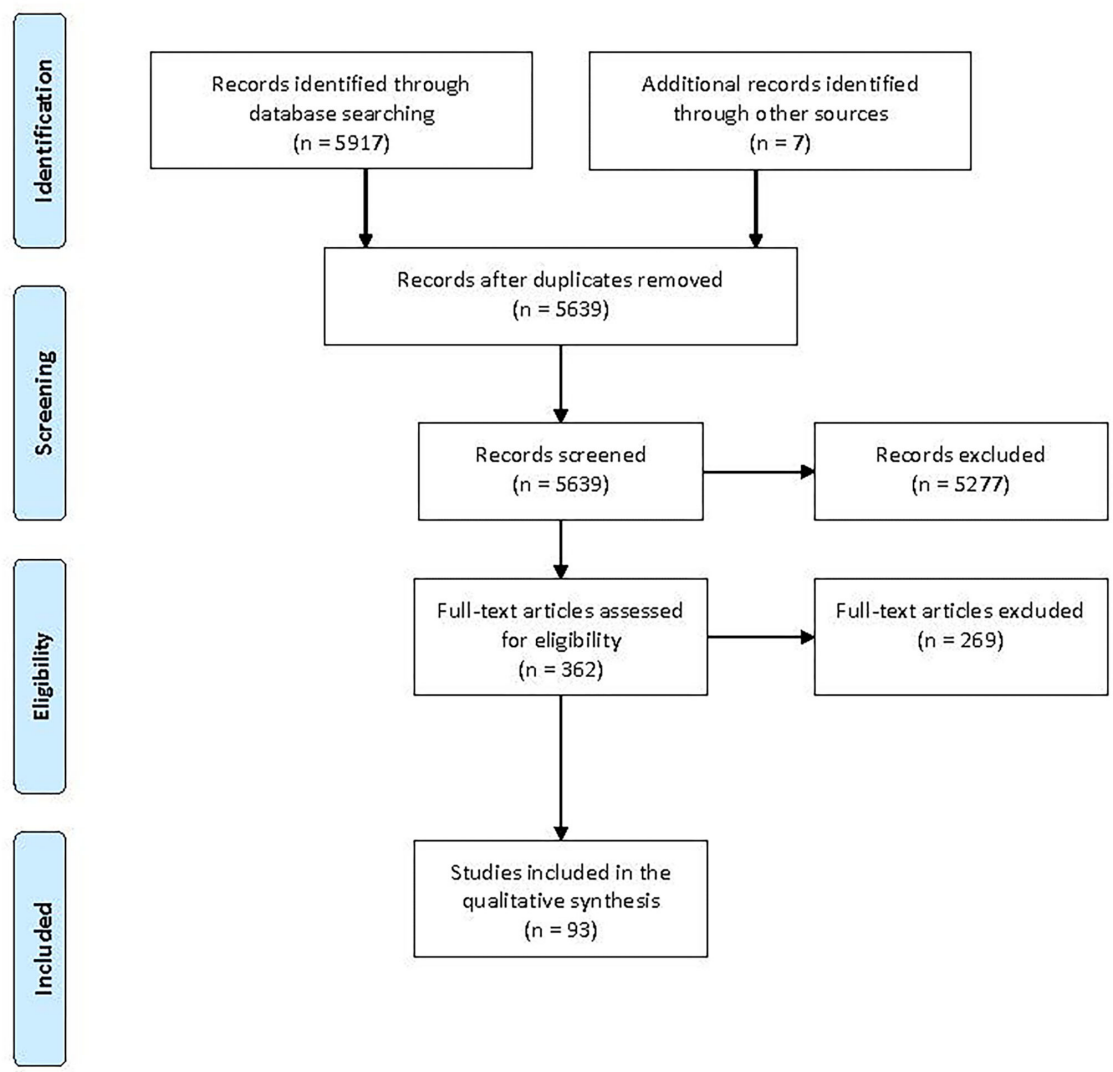
PRISMA flowchart showing the selection of articles.

Working independently, two researchers performed a title and abstract screening. Conflicts were resolved in discussions within the research group. Selected papers then underwent a full text review by two researchers in the same way. Covidence systematic review software (Veritas Health Innovation, Melbourne, Australia) was used for the screening procedure. The search and selection procedure is demonstrated in a PRISMA flowchart (33), see Figure 1.

### Extracting the Evidence

Data related to the objectives were extracted and recorded by the authors individually in a form designed for this study. The following data items were recorded from all included papers:

- Bibliographic details (lead author, title, journal, year, country of origin, full citation).
- A brief narrative description of how the research questions were answered in the paper.
- When applicable, excerpts to illustrate how the question about parental experiences was answered in qualitative papers.

A detailed description of the form and the type of information charted, is published in the *a priori* review protocol (https://zenodo.org/record/3787492#.YBaaUS2HK-s).

### Analysis of the Evidence

Because of the heterogeneity in the material and scanty of research within the review's three objectives, the proposed meta-synthesis in the *a priori* review protocol was dismissed. The three reviewers (AU, MA, ME), working together, examined all the extracted data from the included sources, descriptively mapped, and summarized them aligning the results with the review's three objectives. The qualitative content analysis in this scoping review was descriptive. The data from the included articles were not assessed according to certainty in the results or synthesized [cf. (32)].

This review also considered, extracted, and summarized results from qualitative research studies as well as qualitative data from mixed methods studies. The two primary reviewers (AU, ME), working together, examined all the extracted qualitative findings and grouped these into two themes based on parallels in the parents' experiences of being the deliverers of pain relief. Supported by illustrations from participants' data, the scoping review's qualitative findings were then collated and presented in a narrative form.

### Presentation of the Results

Included studies were organized in three groups aligning the three objectives with the results. Some studies met more than one objective. Evidence was presented in tables and in narrative summaries of the key findings. The results for the three objectives were then discussed in relation to the purpose of this scoping review ending with conclusions and clinical implications.

## Results

### Evidence on the Effectiveness of Parent-Delivered Interventions

The 93 included papers (Figure 1) on parent-delivered interventions are summarized below and presented in tables ordered alphabetically by single interventions followed by combined interventions. The included papers are presented in each table in chronological order to demonstrate how the research fields have evolved.

#### Breastfeeding and Breastmilk

We found 22 papers reporting studies on breastfeeding as pain relief for neonatal pain (Table 1). All but three reported on randomized controlled trials (RCT), years of publication ranged from 2009 to 2020, and most were conducted in Iran and India. The pain-inducing procedures were heel lancing (11 studies), vaccination (eight studies), and venipuncture (three studies). All studies but one showed significant pain-relieving effects on pain scores [Neonatal Infant Pain Scale (NIPS; 12 studies), Premature Infant Pain Profile (PIPP; three studies), Neonatal Facial Coding System (NFCS; two studies), or Douleur Aiguë Nouveau-né (Newborn Acute Pain (DAN); one study)], heart rate (three studies), crying (three studies), or cerebral blood flow (one study). Holsti et al. (35) showed no decrease in Behavioral Indicators of Infant Pain scores but concluded that breastfeeding skills were not affected by the use of breastfeeding during heel lancing.

**Table 1:** Evidence for parent-delivered interventions: breastfeeding.

**Author (year), country**	**Study design**	**Population, setting**	**Type of pain, intervention**	**Key findings**
Leite et al. (34), Brazil	RCT	60 full-term infants, outpatient department	Heel lance, breastfeeding vs. maternal holding	Lower NFCS score and heart rate
Holsti et al. (35), Canada	RCT	57 preterm infants, GA 30–36 weeks, NICU	Heel lance, breastfeeding vs. non-nutritive sucking (soother)	No difference in BIIP score
Del Rey Hurtado de Mendoza et al. (36), Spain	RCT	136 term newborns, tertiary public hospital	Heel lance, SSC mother vs. sucrose vs. SSC + breastfeeding vs. SSC + sucrose	Lower NIPS score in the group with SSC and breastfeeding than in the other groups
Bembich et al. (37), Italy	Pilot study	30 full-term infants^*^	Heel prick, breastfeeding vs. glucose	Lower NIPS score
Lima et al. (38), Brazil	RCT	64 full-term infants, rooming-in	Venipuncture, breastfeeding vs. non-nutritive sucking vs. control (no intervention)	Both breastfeeding and non-nutritive sucking provided lower NIPS score
Modarres et al. (39), Iran	RCT	130 full-term infants, vaccination unit	Vaccination, breastfeeding vs. maternal holding	Lower DAN score
Zhu et al. (40), China	RCT	250 full-term infants, postpartum unit	Heel lance, breastfeeding vs. recorded music vs. combined breastfeeding and recorded music vs. control (no intervention)	Lower NIPS score, longer latency to first cry, shorter duration of first cry with breastfeeding alone and in combination with recorded music
Baskaran (41), India	Cross-sectional study	113 full-term infants, vaccination unit	Vaccination, breastfeeding before painful procedure	Lower NIPS score 30–60 min after breastfeeding
Chiabi et al. (42), Kamerun	RCT	100 full-term infants, maternity unit	Heel prick, breastfeeding vs. glucose	Lower NIPS score than the glucose group
Hashemi et al. (43), Iran	RCT	131 term neonates, vaccination unit	BCG vaccination, swaddling vs. breastfeeding 45 min before vaccination vs. combination swaddling and breastfeeding vs. control (no intervention)	Lower NFCS score in all groups compared to control
Singh et al. (44), India	RCT	60 full-term infants, NICU	Heel lance, breastfeeding vs. maternal holding	Shorter crying time with breastfeeding, less rise in heart rate
Fallah et al. (45), Iran	RCT	120 term neonates, maternity ward	BCG vaccination, breastfeeding vs. SSC mother vs. swaddling	NIPS score was lower than in the SSC and swaddling groups
Zargham-Boroujeni et al. (46), Iran	RCT	75 neonates >34 weeks GA, NICU	Venipuncture, breastfeeding vs. massage on the venipuncture site vs. control (no intervention)	Lower NIPS score than in the control group
Bembich et al. (47), Italy	RCT	80 term newborns, nursery	Heel stick, (1) oral glucose on changing table, (2) maternal expressed breast milk on changing table, (3) maternal holding plus oral glucose, (4) breastfeeding	Lower NIPS score in the holding plus breastfeeding group compared with the other groups
Rioualen et al. (48), France	RCT	102 full-term neonates, maternity ward	Venipuncture, breastfeeding vs. oral sucrose	No difference in NIRS
Gajbhiye et al. (49), India	3-group experiment without randomization	150 full-term infants, postnatal ward	Vaccination, breastfeeding vs. oral sucrose vs. control (no intervention)	Lower PIPP score than in sucrose group
Hatami Bavarsad et al. (50), Iran	RCT	100 full-term infants, maternity ward	Vaccination, breastfeeding vs. expressed breastmilk vs. powdered formula group vs. control group (no feeding)	Lower DAN score than in the other interventions
Soltani et al. (51), Iran	RCT	161 full-term infants, pediatric ward	Heel prick, breastfeeding vs. SSC vs. oral dextrose vs. EMLA cream	The breastfeeding method showed the lowest NIPS score in comparison with the other interventions
Dar et al. (52), Pakistan	RCT	60 full-term infants, outpatient department	Vaccination, breastfeeding vs. control (no intervention)	Shorter crying duration with breastfeeding
Aydin and Inal (53), Turkey	RCT	150 full-term infants, baby nursery	Heel stick, breastfeeding vs. heel warming vs. control (no intervention)	Lowest NIPS score in the breastfeeding group Shorter crying duration with breastfeeding
Kumar et al. (54), India	Observational	300 full-term infants, postnatal ward	Immunization, breastfeeding vs. non-nutritive sucking vs. rocking vs. 25% sucrose vs. distilled water vs. control (no intervention)	Lower DAN score and shorter crying duration in the breastfeeding group compared with controls
Yilmaz and Inal (55), Turkey	RCT	169 term newborn infants, maternity ward	Heel lancing, (1) control group without analgesia, (2) swaddling, (3) swaddling and maternal holding, (4) swaddling and maternal holding and breastfeeding	Lower NIPS score with combined swaddling, maternal holding and breastfeeding compared to all other groups Duration of crying and calming time were shorter with combined swaddling, maternal holding, and breastfeeding than in all other groups

*RCT, randomized control trial; NFCS, Neonatal Facial Coding System; GA, gestational age; NICU, neonatal intensive care unit; BIIP, behavioral indicators of infant pain; SSC, skin-to-skin contact; NIPS, Neonatal Infant Pain Scale; DAN, Douleur Aiguë Nouveau-né; BCG, Bacillus Calmette-Guèrin; NIRS, near-infrared spectroscopy; PIPP, Premature Infant Pain Profile*.

* *Setting not reported in article*.

The use of expressed breast milk was reported in four RCTs from four different countries from 2012 to 2018. None of the studies revealed any significantly better pain relief for heel lancing (two studies), tape removal, or vaccination (one study each) than oral sweet solution, breastfeeding, maternal holding, or SSC. Pain was evaluated with PIPP (three studies) or NIPS (one study) scores (Table 2).

**Table 2:** Evidence for parent-delivered interventions: expressed breastmilk.

**Author (year), country**	**Study design**	**Population, setting**	**Type of pain, intervention**	**Key findings**
Simonse et al. (56), Netherlands	RCT	71 preterm neonates, GA 32–37 weeks, NICU	Heel lance, breast milk (either breastfed or bottle-fed) vs. oral sucrose	No difference in PIPP score between neonates receiving breast milk and those receiving sucrose
Nanavati et al. (57), India	RCT	50 VLBW infants, NICU	Adhesive tape removal, a swab soaked in expressed breast milk was held in the infant's mouth from 2 min before tape removal vs. SSC mother	PIPP score indicated minor or no pain. No difference compared to the SSC group
Bembich et al. (47), Italy	RCT	80 term newborns, nursery	Heel stick, (1) oral glucose on changing table, (2) maternal expressed breast milk on changing table (2 ml), (3) maternal holding plus oral glucose, and (4) breastfeeding	No significant effect on NIPS score for expressed breastmilk compared with maternal holding or glucose
Hatami Bavarsad et al. (50), Iran	RCT	100 full-term infants, maternity ward	Vaccination, expressed breastmilk vs. breastfeeding vs. powdered formula group vs. control group (no feeding)	No significant effect on DAN score compared with the breastfeeding group

*RCT, randomized control trial; GA, gestational age; NICU, neonatal intensive care unit; PIPP, premature infant pain profile; VLBW, very low birth weight; NIPS, Neonatal Infant Pain Scale*.

#### Facilitated Tucking

Facilitated tucking by parents for pain relief was reported in two papers, both from Finland, published in 2006 and 2009. They found lower NIPS score during endotracheal or pharyngeal suctioning and when combined with orally given sucrose lower PIPP and NIPS scores during heel lancing or pharyngeal suctioning (see Table 3).

**Table 3:** Evidence for parent-delivered interventions: facilitated tucking.

**Author (year), country**	**Study design**	**Population, setting**	**Type of pain, intervention**	**Key findings**
Axelin et al. (58), Finland	RCT	20 preterm infants, GA 24–33 weeks, NICU	Endotracheal and pharyngeal suctioning, facilitated tucking vs. control care (no intervention)	Lower NIPS score than no intervention
Axelin et al. (59), Finland	RCT	20 preterm infants, GA 28–32 weeks, NICU	Heel stick and pharyngeal suctioning, facilitated tucking vs. oral glucose vs. opioid (oxycodone) vs. placebo (oral water)	Lower NIPS and PIPP scores

*RCT, randomized control trial; GA, gestational age; NIPS, Neonatal Infant Pain Scale; PIPP, premature infant pain profile*.

#### Holding or Swaddling

Seven studies, published from 2014 to 2020 in Turkey, Iran, Italy (two studies each), and the UK (one study) reported on holding or swaddling by parents (Table 4). Breastfeeding 45 min before vaccination combined with swaddling was reported to lower NFCS score (43), and swaddling alone or in combination with holding lowered NIPS score (55). Except from the latter, when swaddling or holding were single interventions, there was no change in NIPS score (61), electroencephalogram (EEG) activity (31), increased cortical activation (62), or NIPS score and crying time (60).

**Table 4:** Evidence for parent-delivered interventions: holding or swaddling.

**Author (year), country**	**Study design**	**Population, setting**	**Type of pain, intervention**	**Key findings**
Karakoç et al. (60), Turkey	3-group experiment without randomization	120 full-term newborn infants, maternity ward	Blood sampling outer side of left foot, maternal holding on the mothers' laps vs. maternal holding and recorded white noise vs. recorded white noise in crib	Higher NIPS score and longer crying time for the maternal holding group compared with the white noise-only group
Bembich et al. (61), Italy	RCT	40 full-term newborn infants, postnatal ward	Heel prick, mothers holding the dressed infant in their arms vs. oral glucose (on examination table)	Maternal holding was associated with cortical activation in areas associated with the processing of somatic sensations and, in newborns, with affective responses (NIRS). Mother–infant relationship can improve the analgesic effect
Hashemi et al. (43), Iran	RCT	131 term neonates, vaccination unit	BCG vaccination, swaddling vs. breastfeeding 45 min before vaccination vs. combination swaddling and breastfeeding vs. control (no intervention)	Lower NFCS scores than control
Bembich et al. (47), Italy	RCT	80 term newborns, nursery	Heel stick, (1) oral glucose on changing table, (2) maternal expressed breast milk on changing table, (3) maternal holding plus oral glucose, and (4) breastfeeding	Lower NIPS scores in the holding plus breastfeeding group than in the other groups
Jones et al. (31), UK	Observational	27 infants, GA 23–41 weeks, neonatal unit	Heel lance, held by parent with clothing vs. SSC mother vs. control (lying in cot)	No difference in effect on magnitude in noxious-related cortical activity (EEG) compared with lying in cot
Roshanray et al. (61), Iran	RCT	135 full-term newborn infants, health center	Blood sampling, mothers holding the infant in their arms (hug group) vs. massage vs. control (no intervention)	No difference in NIPS score immediately after blood sampling. After 5 min, lower NIPS in mother's hug group compared with the massage and control groups
Yilmaz and Inal (55), Turkey	RCT	169 term newborn infants, maternity ward	Heel lance, (1) control group without analgesia, (2) swaddling, (3) swaddling and maternal holding,and (4) swaddling and maternal holding and breastfeeding	Lower NIPS score with combined swaddling, maternal holding and breastfeeding compared to all other groups Duration of crying and calming time were shorter with combined swaddling, maternal holding, and breastfeeding than in all other groups

*NIPS, Neonatal Infant Pain Scale; RCT, randomized control trial; NIRS, near-infrared spectroscopy; NFCS, Neonatal Facial Coding System; GA, gestational age; EEG, electroencephalogram*.

#### Massage

Parent-delivered massage for neonatal pain relief was reported in two papers from Iran and one each from Turkey and Lebanon from 2012 to 2020 (Table 5). The study on infantile colic showed that weekly crying time was decreased by abdominal massage (63), and two others showed decreased pain from heel stick (PIPP; 39) and venipuncture (NIPS; 40). The remaining study showed no direct effect on NIPS but a lower score 5 min after blood sampling (61).

**Table 5:** Evidence for parent-delivered interventions: massage.

**Author (year), country**	**Study design**	**Population, setting**	**Type of pain, intervention**	**Key findings**
Çetinkaya et al. (63), Turkey	RCT	40 full-term infants, public health clinic	Infantile colic, aromatherapy abdominal massage 5–15 min during colic attacks vs. control (no intervention)	Mean weekly crying time decreased
Abdallah et al. (64), Libanon	Quasi experimental	66 preterm infants, GA 26–36 weeks, NICU	Heel stick, 10 min massage by parents, a minimum of 10 × vs. control (no intervention)	Reduced PIPP score after heel stick
Zargham-Boroujeni et al. (46), Iran	RCT	75 neonates >34 weeks GA, NICU	Venipuncture, massage on the venipuncture site vs. breastfeeding vs. control (no intervention)	Lower NIPS score than breastfeeding and control groups
Roshanray et al. (61), Iran	RCT	135 full-term newborn infants, health center	Blood sampling, massage of the leg and foot 2 min before blood sampling vs. mother's hug vs. control (no intervention)	No difference in NIPS score immediately after blood sampling. After 5 min, lower NIPS in mother's hug group compared with the massage and control groups

*RCT, randomized control trial; GA, gestational age; PIPP, premature infant pain profile; NICU, neonatal intensive care unit; NIPS, Neonatal Infant Pain Score*.

#### Live Parental Infant-Directed Singing

Only one Italian study reported on live parental infant-directed singing for pain relief (65). Maternal live lullaby singing during pregnancy and after birth reduced the incidence of infantile colic in the first month (Table 6).

**Table 6:** Evidence for parent-delivered interventions: live parental infant-directed singing.

**Author (year), country**	**Study design**	**Population, setting**	**Type of pain, intervention**	**Key findings**
Persico et al. (65), Italy	Concurrent cohort study	156 fetus/infants monitored prenatally from 24 weeks GA up to 3 months after birth. Antenatal classes/maternity unit/home	Infantile colic, maternal live lullaby singing during pregnancy and after birth vs. control cohort of non-singing women	In the lullaby-singing cohort, the incidence of infantile colic episodes in the first month was significantly lower than in the control cohort of nonsinging women (concurrent cohort). Infantile colic was reduced in the singing group also in the second month after birth. Maternal singing during pregnancy and after birth could both improve maternal–infant interaction and contribute to preventing neonatal colic

*GA, gestational age*.

#### Skin-to-Skin Contact

Almost half of the included studies, 44 papers, reported on SSC with parents for neonatal pain relief, but only one included fathers in the intervention (66). The papers were published from 2000 to 2020, with nine from the USA, eight from India, seven from Iran, six from Canada, and the rest from various places around the world. Heel stick was the source of pain in 30 of the studies, followed by injections (six studies) and other painful procedures (one or two each). PIPP scores were reduced in 15 studies, but not in five others. Crying duration was reduced in 10 studies and NIPS scores in six. Many studies used more than one outcome variable for pain, and better outcomes were seen in areas such as heart rate, DAN, and NFCS scores, grimacing, and EEG. Some studies, however, showed no better effect for SSC than for other interventions (57, 67, 68) or the control/placebo group (69–71). Soltani et al. (51) found that NIPS scores were higher in the SSC group than in the breastfeeding group.

#### Combined Parent-Delivered Interventions

Many studies have tested parent-delivered interventions combined with other options such as sweet solutions [see, for example, (47, 59, 72)]. Here, we report on eight studies combining two or more parental interventions, all with a randomized controlled design. Two each were performed in Italy and Turkey and one each in Brazil, Canada, Jordan, and Spain. Three studies combined SSC with breastfeeding, two combined breastfeeding and maternal holding, and one combined with rocking with infant-directed speech or singing (enhanced SSC; 43). Four of the studies showed lower pain signs for combined interventions than for single interventions (55, 73–75). Bellieni et al. (76) studied sensorial saturation, an intervention combining touch, massage, taste, voice, smell, and sight, and found that sensorial saturation performed by mothers was as effective as that performed by experienced nurses.

### Parental Experiences of Delivering Pain-Relieving Interventions

Ten studies investigating parents' experiences of delivering pain-relieving interventions were included: four applied qualitative analysis to data from interviews and open-ended questionnaires, three utilized structured questionnaires or instruments, and three used a combination of the abovementioned methods. The qualitative results from the 10 studies are summarized in two themes: *involvement and parental role* and *knowledge and staff support*.

#### Involvement and Parental Role

Parents' opinions about active participation in pain management were unanimous across the included studies. Parents wanted and needed to actively participate in their infant's pain management during painful procedures (10, 12, 14, 58, 77–82). Both mothers and fathers expressed a strong desire to be present and involved during and after a painful procedure in order to comfort their infant, although fathers felt less confident in their ability to alleviate their infant's pain (80). Parents felt they had a vital role in infant pain care, and they wanted as much involvement as possible (80).

In a study by Axelin et al. (58), parents completed a questionnaire on delivering the pain-alleviating intervention *facilitated tucking by parents*. Ninety-five percent reported that although they felt uncomfortable in the situation, they preferred actively delivering the intervention to help comfort their infant during the painful procedure. Parents felt they had an important role in the care of their infant and that their infant was calmer, in less pain, and quicker to calm down when they were involved. Parents also felt their active participation helped them to cope better with their own stress (58).

Skene et al. (14) aimed to explore issues around parental involvement in neonatal pain management but found it so seldom utilized that they had to broaden the question to the more general area of parental participation in comfort care. Only one of the interviewed parents specifically mentioned pain. “At first I didn't consider pain; now when his arms and legs are going, he might be in pain.” (14).

In a study from Kenya, the mothers observed a tendency among healthcare personnel to be more sensitive about providing pain relief when the mothers were present. The mothers described a growing awareness that they could provide verbal soothing during and after painful procedures (10). The mothers said that although witnessing their infants' pain was emotionally traumatic, they wanted to be actively involved to minimize the stress: “You see. It's better to be involved, because after the procedure I would hold my baby and try to calm her down, because after the procedure she is left all alone in pain.” (10).

Facilitators and obstacles to parental involvement are described in articles by Skene et al. (14), Franck et al. (79), Palomaa et al. (12), and Pierrat et al. (82), among others. The physical environment and staff attitudes can be both supportive and hindering factors in parental involvement. “Sometimes [painful procedures] are so routine, doctors and nurses forget they are painful.” (79). “During the procedure was a kind of feeling that another adult's hands do not fit in the incubator at same time.” (12). “The room is comfortable and quiet and spacious. We are allowed to care for our children as much as we want.” (12). “You know they're there if you need them, they can be filling charts but glancing to see how she's doing. You don't feel they're hovering over you and watching your every move. They step back, but not so far that you'd think what if something went wrong.” (14).

The role of the parent in pain management is, according to the included studies, somewhat confusing and ambiguous in many neonatal intensive care units (NICUs) worldwide. If parents perceive the nursing and medical staff as the infants' main caregivers, feeling themselves redundant and unwanted in their infants' pain management or being afraid of failing as a parent or “being in the way” will negatively affect their confidence and competence in parenting (14, 79). “I seem to be a ‘spare part' that has been marginalized.” (79). “I doubt will I be able to do anything; do I know how? It feels that someone else would be better to do pain relief,” and “I believe that my bad feeling reflect to the baby, so I think it is better to be further away when your emotions take too much power.” (12).

Parents' felt confused and frustrated when they felt unable, not allowed, or not encouraged by NICU staff to carry out their preferred role: “I wanted to be present when the cannula was being inserted, but the nurse suggested I leave the room. I left the room feeling upset.” (79).

Parents who are not given the opportunity to comfort their suffering infant can experience increased stress from the losses of their parental role and their ability to protect their infant (80). Being involved in comforting the hospitalized infant can aid in the process of learning to parent (14). Parental involvement in pain management also facilitates the transfer of responsibility from nurse to parent and assists the establishment of attachment behaviors (14). Consequently, there is a need for this parent-focused approach to neonatal pain management, which recognizes not only the importance but also the therapeutic value of parental involvement (14). Parents should be engaged as partners in caregiving and decision making, and they should be given space to assume their role as parent during their infant's hospitalization (12). “We know our own babies best! Use us. ALL we want is to help our children!” (79).

The possibility of alleviating infant pain and stress is a meaningful part of parenting for parents in the NICU (77). However, involvement in pain care must be individualized and tailored to the family's and individual parent's needs and prerequisites (77, 79). “I feel it is important that I'm able to comfort my child with my closeness. The closeness strengthens my motherhood. Unlike when I have to be separated from my child, I'm able to really be with my child in these situations. It really helps.” (77).

Parents who are well-informed and prepared to take an active role in pain care developed a more positive parental role attainment after discharge (78).

#### Knowledge and Staff Support

“I wish I would have had the skill to help her relieve her pain.” (79) Parental involvement in neonatal pain management is closely linked to the parent's knowledge of pain and to the culture of care on the unit (82). Parents have consistently stated in the research that they want and need more information and knowledge about neonatal pain management. This is also the common denominator in the qualitative data from the studies included in this scoping review: “Explain to parents what we can do to help our babies, tell us what signs to look for that the baby is in pain and perhaps offer on admission a group meeting to help new parents understand the management and policies of pain relief in that hospital.” (79).

“Maybe talk to mothers and educate them on the benefits of those strategies. // But if you can talk to them and tell them if you place the baby in this position the baby will calm down and will sleep. Because it is not easy to get it without being explained to its benefits.” (10).

To feel truly involved and actively deliver pain management to their infant, parents want to be prepared in advance and educated about the effectiveness of various parent-delivered methods and how to apply them. To this end, they need information and preparation at the appropriate time. “Parents should be told in advance of how to relieve pain or calm babies when they feel pain (especially when they undergo procedures, e.g., taking blood). Giving advice during or after procedures is sometimes too late.” (79).

Other important factors in parental involvement in pain management are parental counseling and support from NICU staff. Nurses' knowledge and attitudes toward parent-delivered pain management play a critical role in facilitating change in the NICU (12). “Nurses' encouragement of parental involvement in comfort care facilitated parental proximity, parent/infant reciprocity, and parental sense of responsibility” (14). “To be respected as the baby's parent, to be fully informed and given choices and to be aware of what works best for baby in my role of managing baby—be it talking, holding or calming baby and to be supported or told by staff ‘Thanks, your help helped your baby'.” (79).

Nurses should encourage parents to actively participate in pain management and show parents how to use parent-delivered interventions (77). The knowledge transfer should be both collaborative and bidirectional; parents are the experts on their own infants and should guide the staff about the individual needs of the infant and parent (14).

The need for individually adapted information, instructions, and support in various formats was also raised in the results from the quantitative surveys with parents (80–82). Just as involvement in pain care must be individualized and tailored to the parent's needs and prerequisites, information about parent-delivered methods must also be offered sensitively (80, 81). Parents' readiness and receptivity to learning about infant pain must be monitored, since some parents want to know all there is to know, but others might benefit more from small and selective bits of NICU information (80). Health care professionals may also need more training in transfering knowledge about parent-delivered interventions to diverse families with very different needs and capabilities (82). The family-centered approach in NICUs adopts a culture of collaboration between parents and professionals, which has been highlighted as improving infants' pain management (82). Leadership and staff attitudes and beliefs have been shown to play an important role in parents' successful involvement in neonatal pain management, and the presence of a local champion, often a nurse, whose duty is to facilitate the implementation of pain control measures, is the main reported factor in closing the knowledge-to-practice gap in neonatal pain care (82).

### Recommendations in Clinical Guidelines

Eight guidelines recommending parental neonatal pain alleviation issued by a national or international authority or a professional organization or network were included (**Table 9**). The guidelines originated in Italy, Australia, Sweden, the USA, the UK, and France.

The Italian Society of Neontology pain study group (83) recommended specific pain-relieving measure procedures with grades based on the level of evidence. Breastfeeding and breast milk were graded at the highest level of evidence and were therefore highly recommended. The guideline graded parental presence with the lowest level of evidence and did not distinguish between passive or more active involvement. The same grading system was used by the Association of Pediatric Anesthetists of Great Britain and Ireland (84), which highly recommended breastfeeding, holding or swaddling, massage, and SSC.

Evidence and recommendations for breastfeeding and for SSC were presented in six of the included guidelines. The American Academy of Pediatrics (85) had recommendations only for SSC, while the other guidelines included recommendations for more than one intervention, predominantly breastfeeding.

Fewer guidelines recommended facilitated tucking (three guidelines), holding or swaddling (three guidelines), combined parent-delivered interventions (two guidelines), and massage (two guidelines). No guidelines to date include recommendations for live parental infant-directed singing.

## Discussion

Since Anand and Hickey (86) showed the importance of newborn pain management in the late 1980s, researchers and clinicians have struggled to provide sufficient pain relief for the frequent painful, though usually life-saving, procedures inflicted on vulnerable infants in the NICU (87). Many steps remain to be taken before all infants are provided adequate pain relief, as highlighted in the report of the International Lancet Child & Adolescent Health Commission (2). This knowledge-to-practice gap is also highlighted in this scoping review. Research into the efficacy of parent-delivered pain management in neonatal care is growing, but evidence remains scanty for interventions other than SSC. Of the included studies, most research on parent-delivered pain interventions was conducted in the last decade (2010–2020) and has accelerated in the last 5 years (2015–2020). Of 76 relevant articles, 58% (*n* = 44) dealt with SSC (2000–2020) and 29% (*n* = 22) with breastfeeding (2009–2020). Only 10% (*n* = 8) reported on combined parent-delivered methods (2007–2020). The results of this scoping review and other systematic reviews (27, 88) clearly show sufficient evidence for the efficacy of breastfeeding and SSC, alone or preferably in combination. Further evidence is unlikely to change our estimation of the pain-relieving effects of these methods. To make infant pain better and more visible, we therefore urge, supported by the guidelines, a global consensus on, and amplification and expansion of, the parent-delivered interventions breastfeeding and SSC.

Parents' role in the pain experience of older children has received considerable attention (11), but previous research has shown little interest in NICU parents' expectations about their infant's pain (8). Parents have consistently stated in studies that they wish to remain with their infant during painful procedures, but they feel unsupported in taking an active role (e.g., 8, 16, 54, 58). Being present when staff provides pain management and being actively involved in delivering the pain relief are two separate things with distinct outcomes in efficacy (Tables 1, 7, 8). The qualitative interview data and quantitative surveys included in this scoping review show that parents want and need to actively participate in their own infant's pain management during painful procedures (10, 12, 14, 58, 77–82). The fact that most parents' wish to take an active role in helping their infant manage procedural pain is also confirmed in systematic reviews (e.g., 16). However, as shown in this review, very few studies have been able to investigate parents' experiences of delivering pain management for their own infant. Studies on parents' active participation in infant pain management and their views on being a mediator in their own infant's pain relief are scarce. One reason for this could be that very few units today actively support and facilitate parent-delivered pain management.

**Table 7:** Evidence for parent-delivered interventions: skin-to-skin contact.

**Author (year), country**	**Study design**	**Population, setting**	**Type of pain, intervention**	**Key findings**
Gray et al. (89), USA	RCT	30 full-term infants, maternity ward	Heel lance, SSC mother vs. control (swaddled in crib)	Crying and grimacing were reduced by 82 and 65%, respectively
Ludington-Hoe et al. (90), USA	RCT	24 premature infants, NICU	Heel stick, SSC mother vs. incubator care	Heart rate and crying responses to pain were significantly reduced
Castral et al. (91), Brazil	RCT	59 premature infants, GA 30–36 weeks, NICU	Heel prick, SSC mother vs. incubator care	Infants who received skin-to-skin contact were more likely to have lower NFCS scores. Changes in crying time and heart rate were less for the treated infants
Freire et al. (92), Brazil	RCT	95 preterm infants, GA 28–36 weeks, NICU	Heel lance, SSC mother vs. control (no intervention)	Lower PIPP score
Johnston et al. (93), Canada	Randomized crossover	61 preterm neonates, GA 28–31 weeks, NICU	Heel lance, SSC mother vs. control (swaddled in incubator)	Lower PIPP score, shorter recovery time
Kostandy et al. (94), USA	Pilot study, randomized crossover	10 premature infants, GA 30–32 weeks, NICU	Heel stick, SSC mother vs. incubator care	Reduced crying time
Cong et al. (95), USA	Pilot study, randomized crossover	14 preterm infants, GA 30–32 weeks, NICU	Heel stick, SSC mother vs. incubator care	More autonomic stability
Johnston et al. (67), Canada	Randomized crossover	90 preterm infants, GA 32–36 weeks, NICU	Heel lance, SSC mother vs. enhanced SSC with rocking, infant-directed speech/infant-directed singing and sucking	PIPP score in both groups in the minor pain strata but no difference between groups
Cong et al. (96), USA	Randomized crossover	18 + 10 preterm infants, GA 30–32 weeks, NICU	Heel stick, SSC mother 80 or 30 min vs. incubator care	30 min SSC reduced PIPP score and cortisol levels
Fernandes et al. (97), Portugal	RCT	110 preterm infants, GA 28–36 weeks, NICU	Venipuncture, SSC mother + sucrose and pacifier vs. sucrose and pacifier	Reduced grimacing
Saeidi et al. (98), Iran	RCT	60 full-term newborns, maternity ward	Vaccination, SSC mother vs. control (infant wrapped in a blanket and put near the bed of the mother)	Lower NIPS score and crying duration
Cong et al. (99), USA	Case study	2 preterm twins, GA 28 weeks, NICU	Heel stick, SSC mother for 15 or 30 min vs. incubator care	Lower PIPP score and shorter crying time, better autonomic stability
Cong et al. (100), USA	Randomized crossover	26 preterm infants, GA 28–32 weeks, NICU	Heel stick, SSC mother for 15 or 30 min vs. incubator care	Better autonomic stability
Del Rey Hurtado de Mendoza et al. (36), Spain	RCT	136 term newborns, tertiary public hospital	Heel lance, SSC mother vs. sucrose vs. SSC + breastfeeding vs. SSC + sucrose	Lower NIPS score in the group with SSC and breastfeeding than in the other groups
Johnston et al. (101), Canada	Randomized crossover	18 preterm neonates, GA 28–37 + 2, NICU	Heel lance, SSC mother vs. SSC unrelated alternative female	Non-related women are marginally less effective than mothers at decreasing pain response
Memarizadeh et al. (102), Iran	RCT	20 premature infants, GA 27–36 weeks, NICU	Heel stick, SSC mother vs. incubator care	Lower PIPP score
Kostandy et al. (103), USA	RCT	36 full-term infants, postpartum unit	Vaccine, SSC mother vs. control (lying in cot)	Shorter crying time during recovery
Mitchell et al. (69), USA	RCT	38 preterm infants, GA 27–30 weeks, NICU	Tracheal or nasal suctioning, SSC over 5 days vs. standard care in incubator	No significant difference in cortisol
Nanavati et al. (57), India	RCT	50 WLBV infants, NICU	Adhesive tape removal, SSC mother vs. expressed breast milk	PIPP score indicated minor or no pain. No difference compared to expressed breast milk group
Nimbalkar et al. (104), India	Randomized crossover	50 preterm neonates, GA 32–36 weeks, NICU	Heel prick, SSC mother vs. control (swaddled in cot)	Lower heart rate and PIPP score
Pasquier et al. (105), Canada	RCT	60 full-term infants after cesarian section, delivery unit	Vitamin K injection, SSC mother vs. support and monitoring (control)	Lower NIPS score, smaller variations in salivary cortisol
Campo et al. (70), Phillipines	RCT	31 full-term infants, maternity unit	Heel prick, SSC mother vs. mothers holding the dressed infant in their arms	No significant difference in HR, SaO_2_, or NIPS score
Chidambaram et al. (106), India	Crossover	100 preterm infants, GA 32–26 weeks, NICU	Heel prick, SSC mother vs. control (no intervention)	Lower PIPP score
Mosayebi et al. (107), Iran	Randomized crossover	64 preterm infants, GA 30–36 weeks, NICU	Heel lance, SSC mother vs. incubator care (swaddled)	Lower PIPP score
Gao et al. (108), China	RCT	75 preterm infants, GA <37 weeks, NICU	Repeated heel stick, repeated SSC mother vs. incubator group	Lower heart rate, shorter crying time and facial grimacing. Stable effect over repeated heel sticks
Padhi et al. (71), India	Prospective pilot study	20 premature infants, mean GA 30.8 weeks, NICU	Eye examination, reversed SSC mother	Significantly lower change in RR during reversed SSC
Liu et al. (109), China	RCT	40 full-term newborns, obstetric ward	Heel stick, SSC mother vs. control (wrapped in clothes)	Reduced DAN score, lower HR, better SaO_2_ and shorter crying time
Rad et al. (110), Iran	Case-control	55 newborn infants, 15–60 days old, children's clinic	Infantile colic, SSC mother at home at least 2 h/day vs. no intervention	Reduced restlessness and fussiness
Choudhary et al. (111), India	Crossover	140 preterm infants GA <37 weeks, NICU	Heel lance, SSC mother vs. no intervention	Shorter duration of cry, lower PIPP score
Dezhdar et al. (112), Iran	RCT	90 preterm infants, GA >37 weeks, NICU	Venipuncture, SSC mother vs. swaddling vs. no intervention group (control)	Lower PIPP score 60 s after venipuncture
Hoxha et al. (113), Albania	RCT	40 term infants, NICU	Heel lance, SSC mother vs. sucrose vs. non-nutritive sucking vs. no intervention group (control)	Shorter cry duration, lower HR and RR, and higher SaO_2_
Leite et al. (75), Brazil	RCT	55 full-term newborns, maternity ward	Hepatitis B vaccination, SSC vs. breastfeeding with SSC	Breastfeeding in combination with SSC showed lower HR than breastfeeding alone. Lower NFCS after the injection
Olsson et al. (114), Sweden	Randomized crossover	10 premature infants, GA 26–35 weeks, NICU	Venipuncture, SSC mother with oral glucose vs. lying in cot with oral glucose	Lower increase in NIRS variables
Seo et al. (115), South Korea	Unclear	56 full-term infants, nursery	Heel stick, SSC mother vs. control (no intervention)	Lower PIPP score, shorter duration of crying, lower HR
Fallah et al. (45), Iran	RCT	120 term neonates, maternity ward	BCG vaccination, SSC mother vs. breastfeeding vs. swaddling	NIPS score was lower with breastfeeding than in the SSC and swaddling groups
Ferrara et al. (116), Uganda	Pilot study	131 full-term infants, maternity ward	Vitamin K injection, SSC mother vs. routine care (examination table)	Bigger proportion had low NIPS score
Murmu et al. (117), India	Crossover	51 preterm neonates, GA 30–36 weeks, NICU	Heel lance, SSC mother vs. SSC alternative female vs. swaddling	Lower PIPP score in both groups compared to swaddling
Shukla et al. (68), India	RCT	100 preterm neonates, GA 29–36 weeks, NICU	Heel stick, SSC mother vs. sucrose	No difference in PIPP score compared with sucrose
Soltani et al. (51), Iran	RCT	161 full-term infants, pediatric ward	Heel prick, SSC vs. breastfeeding vs. oral dextrose vs. EMLA cream	Higher NIPS score than in the breastfeeding group
Hurley et al. (118), Canada	RCT	242 preterm infants, GA <37 weeks, NICU	Heel lance, SSC mother vs. SSC with sucrose vs. sucrose	No difference in PIPP score compared with sucrose
Kristoffersen et al. (66), Norway	RCT	35 preterm infants, GA <32 weeks, NICU	Eye examination, SSC mother/father vs. standard care with supportive positioning by parents	No difference in PIPP score
Campbell-Yeo et al. (119), Canada	RCT	242 preterm infants, GA <37 weeks, NICU	Heel stick, SSC mother vs. sucrose vs. SSC mother and sucrose combined	No difference in PIPP score compared with sucrose
Jones (2020), UK	Observational	27 infants, GA 23–41 weeks, neonatal unit	Heel lance, SSC mother vs. held by parent with clothing vs. control (lying in cot)	Reduced magnitude in noxious-related cortical activity (EEG) more than did holding or no intervention
Nimbalkar et al. (120), India	RCT	100 preterm neonates, GA 28–36 weeks, NICU	Heel stick, SSC mother vs. sucrose	No difference in PIPP score compared with sucrose

*RCT, randomized control trial; SCC, skin-to-skin contact; NICU, neonatal intensive care unit; GA, gestational age; NFCS, Neonatal Facial Coding System; PIPP, premature infant pain profile; NIPS, Neonatal Infant Pain Score; VLBW, very low birth weight; HR, heart rate; SaO_2_, oxygen saturation; RR, respiration rate; DAN, Douleur Aiguë Nouveau-né; EEG, electroencephalogram*.

**Table 8:** Evidence for parent-delivered interventions: combined parent-delivered interventions.

**Author (year), country**	**Study design**	**Population, setting**	**Type of pain, intervention**	**Key findings**
Bellieni et al. (76), Italy	RCT	66 full-term infants, nursery	Heel prick, (1) sensorial saturation without perfume performed by nurses, (2) sensorial saturation without perfume performed by mothers, and (3) glucose plus sucking	Sensorial saturation performed by mothers as effective as that performed by experienced nurses
Johnston et al. (67), Canada	Randomized crossover	90 preterm infants, GA 32–36 weeks, NICU	Heel lance, SSC mother compared with enhanced SSC with rocking, infant-directed speech/infant-directed singing, and sucking	PIPP scores in both groups in the minor pain strata, but no between- group differences
Okan et al. (121), Turkey	RCT	107 full-term infants, maternity ward	Heel lance, (1) breastfeeding with SSC combined with touching the infants' heads and backs and talking to the infant whenever the mothers wished, (2) maternal holding with SSC but no breastfeeding, and (3) no-contact group with the infants lying on an examination table	HR, SaO_2_, and length of crying were significantly lower in groups 1 and 2 than in group 3, but no difference found between groups 1 and 2
Marín Gabriel et al. (73), Spain	RCT	136 full-term infants, maternity ward	Heel lance, (1) breastfeeding and SSC, (2) oral sucrose and SSC, (3) SSC alone, and (4) oral sucrose alone	Lower NIPS score in the breastfeeding and SSC group compared with other groups
Obeidat et al. (74), Jordan	RCT	128 full-term infants, maternity ward	Heel lance, (1) breastfeeding combined with maternal holding and (2) maternal holding, on mothers' lap, alone	Lower PIPP score with combined breastfeeding and maternal holding
Leite et al. (75), Brazil	RCT	55 full-term newborns, maternity ward	Hepatitis B vaccination, (1) SSC and (2) breastfeeding with SSC	Breastfeeding in combination with SSC showed lower HR than breastfeeding alone; lower NFCS after the injection
Bembich et al. (47), Italy	RCT	80 full-term newborns, nursery	Heel stick, (1) oral glucose on changing table, (2) maternal expressed breast milk on changing table, (3) maternal holding plus oral glucose, and (4) breastfeeding	Different cortical patterns (NIRS) were evoked in the four groups Glucose and breast milk are more effective when combined with the maternal–infant relationship than when given alone
Yilmaz and Inal (55), Turkey	RCT	160 full-term infants, maternity ward	Heel lance, (1) control group without analgesia, (2) swaddling, (3) swaddling and maternal holding, and (4) swaddling and maternal holding and breastfeeding	Lower NIPS score with combined swaddling, maternal holding and breastfeeding compared to all other groups Duration of crying and calming time were shorter with combined swaddling, maternal holding, and breastfeeding than in all other groups

*RCT, randomized control trial; GA, gestational age; NICU, neonatal intensive care unit; SSC, skin-to-skin contact; PIPP, premature infant pain profile; HR, heart rate; SaO_2_, oxygen saturation; NIPS, Neonatal Infant Pain Score; NFCS, Neonatal Facial Coding System; NIRS, near-infrared spectroscopy*.

The parents' actual voices are also overlooked, under-utilized, and under-studied as basic resources in neonatal pain management. The musical qualities of the mother's voice are salient in the perinatal experience of speech, enculturation, and attachment. The mother's voice is a multisensory and multimodal event both prenatally and after birth. In pain studies investigating parent-delivered interventions, the involved parents are quiet, even deliberately silenced, or the parents' vocal and musical engagement with their infants is not systematically reported (e.g., 43). Live infant-directed singing is a relationship-based communication tool for parents in regulating the infant's state, affects, and arousal levels (122). The soothing, comforting, and emotion-regulating properties of a lullaby are well-known cross-culturally and historically (122, 123). Research shows that infant-directed singing is more effective than infant-directed speech in lowering infants' elevated arousal levels and ameliorating distress (123). A parent's live lullaby singing is directly attuned to the moment-to-moment biopsychosocial experience of the infant during the painful situation. Live infant-directed singing provides a down-regulating, real-time arousal regulator for the infant to attune to, communicating a shared affect and empathy. Live parental infant-directed singing is therefore something to consider as an adjuvant in the control of infant pain, but more research is needed to confirm its effectiveness.

In making infant pain better and more understood, future research should make the parents visible and audible in infant pain management and make their experiences of delivering infant pain management important. Infant-focused quantitative research in which the infant, and to some extent the parent, are viewed more or less as victims of painful procedures must adopt a more family-integrated biopsychosocial approach using mixed research methods to capture the active role of the infant–parent dyad in managing procedural pain. This scoping review highlights the already sufficient evidence of parents' needs and desires to deliver pain alleviation. Further studies are needed to better understand the parents' motivational factors for engaging in pain management and their emotions surrounding this. There is also enough evidence for the importance of implementing timely, individualized, preparatory knowledge transfer in parent-delivered pain management. Nurses' knowledge and attitudes toward parent-delivered pain management play a critical role in supporting parents' in their caregiver roles and facilitating change in the NICU to make infant pain better. The ongoing COVID-19 pandemic also puts infant-parent interaction at risk, due to fear of infection transmission that can lead to separation of the infant and the parent(s). However, Tran and colleagues (124) concludes that based on currently available data, prolonged skin-to-skin contact and early exclusive breastfeeding should still be used, as the best strategy for neonatal care under the pandemic.

New guidelines on family-centered or family-integrated care are introduced regularly, but most lack recommendations on infant pain management and consequently on parents' active involvement in infant pain care. Currently, few guidelines anywhere recommend neonatal pain alleviation delivered by parents (Table 9), and few or none also consider recommendations for parent-delivered interventions in low-income and low-tech settings, where these interventions could be cost-effective and simple to implement (126). To make infant pain matter, global, national, and local guidelines must start acknowledging neonatal pain and parent-delivered pain management, show the current evidence for various parent-delivered interventions, and recognize the gap in evidence for promising parent-delivered methods such as massage and live parental infant-directed singing. Guidelines also lack important updates for combined parent-delivered pain management. Research shows that a combination of several non-pharmacological interventions increases the analgesic effect (20). Neonatal pain research suggests that combined parent-delivered interventions, especially multisensory strategies such as SSC and breastfeeding, deliver synergistic effects (30, 75). This scoping review confirms the synergistic effects of combined parent-delivered interventions on infants' behavioral pain responses (Table 8). Often, interventions are used not in isolation, but concurrently. The combination of the parent's voice, skin, warmth, breathing rhythm, taste, and scent fully match and harmonize with the infants' multisensory, biopsychosocial state of being. However, more research in combined parent-delivered interventions, research that also includes relationship-based interventions such as the parent's attuned live singing in parent-delivered pain management, is obviously necessary (130).

**Table 9:** Recommendations in clinical guidelines.

**Author (year), country**	**Issued by**	**Breastfeeding**	**Breast milk**	**Facilitated tucking**	**Holding or swaddling**	**Massage**	**Live parental infant-directed singing**	**Skin-to-skin contact**	**Combined parent-delivered interventions**
Lago et al. (83), Italy	Pain study group of the Italian Society of Neontatology^a^	A	A	C	Maternal touching and holding D, swaddling C			B	Sensorial saturation: B
Spence et al. (125), Australia	The Australian and New Zealand Neonatal Network^b^	I	I						
Nyqvist et al. (126), Sweden	First European Conference and Seventh International Workshop on Kangaroo Mother Care								x
Academy of Breastfeeding Medicine (127), USA	The Academy of Breastfeeding Medicine Protocol Committee	x	x					x	
Howard et al. (84), UK	Association of Pediatric Anesthetists of Great Britain and Ireland^a^	A			A	A		A	
Baley (85), USA	American Academy of Pediatrics							x	
Keels et al. (128), USA	American Academy of Pediatrics	x	x	x		x			x
Roue et al. (129), France	The European Network on Early Developmental Care	x		x	x			x	

*The guidelines do not state whether parents or staff provide the pain-alleviating intervention. ^a^Level of evidence and grades of recommendation ranging from A to D based on the Scottish Intercollegiate Guidelines Network classification. A represents the highest and D represents the lowest grade of recommendation. ^b^Levels of evidence and quality of evidence according to the National Health and Medical Research Council where I means evidence obtained from a systematic review. x denotes the intervention is suggested in the guidelines*.

## Strengths and Limitations

The authors acknowledge the limitations to this review in accordance to the search strategy which tried to balance feasibility with breadth and comprehensiveness to include relevant quantitative, qualitative and mixed methods studies. As with any scoping review, it is possible that the search and inclusion strategy and especially limitations related to language and years led to omission of research. The manual searches and the authors' expertise in this field are believed to have balanced these limitations. The objectives of this review were devised to cover various aspects of parent-delivered pain management which resulted in a purposely wide search syntax with a high bias error in the search. However, this bias was managed in the subsequent systematic and exhaustive manual assessment of all studies. The scoping review's three objectives were challenging and time consuming to target with the divergent search structures in the databases and the search was therefore limited to five databases which typically capture research within the chosen topic. A search in Cochrane Database of Systematic Reviews could further have strengthened our findings, but we believe that very few articles have been missed, in the search of the other databases. The manual searches for studies beyond the database search were intended to adjust for this.

This scoping review did not include unpublished research or gray literature. Peer-reviewed literature was determined as the basic criteria for the included evidence in this review since this scoping review did not attempt to undertake quality appraisal of the included studies or a statistical synthesis of the effectiveness of the results. Synthesis of quantified effects or qualitative content analysis would have been challenged by the diversity in study designs and interventions, and the large range of reporting methods used within the studies as well as scarcity of studies on parents' active participation in infant pain management and their views on being a mediator in their own infant's pain relief. However, a meta-analysis or interpretive qualitative analysis is generally not required in scoping reviews. This comprehensive review is well-timed. The foremost strength is the topic itself targeting an under-studied but vital area of neonatal pain management, which may be of great interest to a general audience. The results of this scoping review might hopefully incite transformative changes on all levels in the care of the newborn infant.

## Conclusion

There currently remains a knowledge-to-practice gap in parent-delivered management of infants' procedure-related pain. This scoping review highlights the many advantages of involving parents in pain management for the benefit not only of the infant and parent, but also in the interest of health care. This paper presents evidence for the efficacy of SSC and breastfeeding, preferably in combination. These parent-delivered interventions are safe, valid, and ready for prompt introduction in infants' pain care globally. Among other non-pharmacological approaches, the biopsychosocial perspective strongly supports parent-delivered interventions in which the parent herself/himself is a mediator of pain relief, which is consistent with modern family-integrated care. Yet, we do not know enough about parents' motivational factors in, and experiences of, delivering pain alleviation, but we do know that parents want and need to actively participate in their infant's pain management and that they should be sensitively informed by the NICU staff about how to apply parent-delivered methods. More research on combined parent-delivered interventions, including the communicative and relational aspects of parent-delivered pain alleviation such as the parent's voice and her/his musical presence, is needed to advance infant pain care. More guidelines in this field also need to update the knowledge they disseminate and include infant pain management, parent-delivered pain methods, and the synergistic effects of combining these interventions. They should also address parent involvement in low-income and low-tech settings. This scoping review may serve as a starting point to help close the knowledge-to-practice gap in parent-delivered neonatal pain management and we hope helps make infant pain matter, make it understood, make it visible, and make it better.

## Data Availability Statement

The original contributions presented in the study are included in the article/supplementary material, further inquiries can be directed to the corresponding author/s.

## Author Contributions

AU and ME designed the study. AU, MA, and ME performed screening and data extraction, analyzed the material, and contributed to the writing of the manuscript. All authors approved the final version.

## Conflict of Interest

The authors declare that the research was conducted in the absence of any commercial or financial relationships that could be construed as a potential conflict of interest.
